# TWEAK/Fn14 Signaling Modulates Goblet Cell-Associated Mucin Responses in Mouse Small Intestinal Epithelial Organoids

**DOI:** 10.3390/cells15171541

**Published:** 2026-08-26

**Authors:** Shun Tamaki, Masayuki Kono, Sakura Arikawa, Yui Higashi, Tatsunori Maekawa, Yusuke Hara, Taizo Tsujimoto, Fumitaka Kawakami, Motoki Imai, Yoshifumi Kurosaki, Shokichi Naito, Naohito Ishii, Takafumi Ichikawa, Rei Kawashima

**Affiliations:** 1 Department of Regulation Biochemistry, Graduate School of Medical Sciences, Kitasato University, Sagamihara 252-0373, Kanagawa, Japan; tamaki.shun@kitasato-u.ac.jp (S.T.); kono.masayuki@st.kitasato-u.ac.jp (M.K.); arikawa.sakura@st.kitasato-u.ac.jp (S.A.); higashi.yui@st.kitasato-u.ac.jp (Y.H.); maekawa@kitasato-u.ac.jp (T.M.); yhara@insti.kitasato-u.ac.jp (Y.H.); tsujimoto.taizo@kitasato-u.ac.jp (T.T.); kawakami@kitasato-u.ac.jp (F.K.); kuro96@kitasato-u.ac.jp (Y.K.); naohito3@ahs.kitasato-u.ac.jp (N.I.); t.ichika@kitasato-u.ac.jp (T.I.); 2 Department of Biochemistry, School of Allied Health Sciences, Kitasato University, Sagamihara 252-0373, Kanagawa, Japan; 3 Regenerative Medicine and Cell Design Research Facility, School of Allied Health Sciences, Kitasato University, Sagamihara 252-0373, Kanagawa, Japan; imai-m@kitasato-u.ac.jp; 4 Clinical Research Advancement Section, National Institute of Global Health and Medicine, Japan Institute for Health Security (JIHS), Shinjuku 162-8655, Tokyo, Japan; 5 Department of Gastroenterology, Graduate School of Medical Sciences, Kitasato University, Sagamihara 252-0373, Kanagawa, Japan; 6 Department of Gastroenterology, School of Medicine, Kitasato University, Sagamihara 252-0373, Kanagawa, Japan; 7 Department of Health Administration, School of Allied Health Sciences, Kitasato University, Sagamihara 252-0373, Kanagawa, Japan; 8 Department of Applied Tumor Pathology, Graduate School of Medical Sciences, Kitasato University, Sagamihara 252-0373, Kanagawa, Japan; 9 Department of Molecular Diagnostics, School of Allied Health Sciences, Kitasato University, Sagamihara 252-0373, Kanagawa, Japan; 10 Clinical Chemistry Laboratory, Department of Medical Laboratory Sciences, School of Allied Health Sciences, Kitasato University, Sagamihara 252-0373, Kanagawa, Japan; 11 Department of Nephrology, Graduate School of Medical Sciences, Kitasato University, Sagamihara 252-0373, Kanagawa, Japan; snaito@med.kitasato-u.ac.jp; 12 Department of Nephrology, School of Medicine, Kitasato University, Sagamihara 252-0373, Kanagawa, Japan

**Keywords:** TWEAK/Fn14 signaling, goblet cells, mucin, MUC2, TNF, small intestinal epithelial organoids, mucus barrier, intestinal inflammation

## Abstract

**Highlights:**

**What are the main findings?**

- Fn14 was expressed in MUC2-positive cells in intestinal tissue and showed membrane-associated staining in small intestinal epithelial organoids.
- TWEAK treatment alters Muc2 mRNA expression and mucin-associated features, with possible involvement of TNF signaling.

**What are the implications of the main findings?**

- These findings show that TWEAK treatment is associated with goblet cell-related mucin responses, with possible involvement of TNF signaling.
- This organoid-based model provides a platform for studying epithelial regulation of mucus barrier-related processes under inflammatory signaling conditions.

**Abstract:**

Intestinal barrier dysfunction and mucus layer abnormalities are central features of inflammatory bowel disease, yet the epithelial mechanisms regulating goblet cell function remain incompletely understood. The TWEAK/Fn14 pathway is involved in intestinal inflammation, but its role in small intestinal epithelial responses, particularly in goblet cells, has not been fully clarified. In this study, we investigated TWEAK/Fn14 signaling using mouse small intestinal epithelial organoids. Fn14 was expressed in MUC2-positive cells in intestinal tissue, with staining preferentially observed toward the mucin-facing region. Fn14 staining was also observed in small intestinal epithelial organoids. TWEAK stimulation increased *Tnf* mRNA expression by approximately 1.56-fold and reduced *Muc2* mRNA expression to approximately 66% of control, without markedly affecting epithelial proliferation or stem cell markers. TWEAK did not significantly alter the PAS-positive mucin area or AB-PAS-defined mucin composition, whereas PGM34 staining showed a non-significant tendency to increase. TNF treatment reproduced some of the TWEAK-associated changes, including reduced *Muc2* mRNA expression and alterations in mucin-associated parameters, suggesting that TNF may contribute to some of the epithelial responses associated with TWEAK treatment. Treatment with an anti-TNF neutralizing antibody modified several TWEAK-associated responses, including changes in epithelial-associated gene expression and mucin-associated staining parameters. These findings suggest that TWEAK treatment is associated with goblet cell-related mucin responses in mouse small intestinal epithelial organoids, with possible involvement of TNF signaling.

## 1. Introduction

Inflammatory bowel disease (IBD) is increasing worldwide, and disruption of the intestinal epithelial barrier and abnormalities in the mucus layer are recognized as central features of its pathophysiology [1,2,3,4,5]. The intestinal mucus layer, which is primarily composed of mucins secreted by goblet cells, prevents direct contact between the epithelium and luminal bacteria and is essential for maintaining intestinal homeostasis [6,7]. Accordingly, impaired goblet cell function and altered mucin production may contribute to the development and progression of intestinal inflammation, highlighting the importance of understanding the molecular mechanisms that regulate goblet cell-associated mucus barrier responses. Beyond changes in overall mucin abundance, mucin expression patterns can also reflect epithelial phenotype within the gastrointestinal tract. In gastric lesions and intestinal metaplasia, MUC2 has been associated with an intestinal mucin phenotype, whereas MUC5AC is associated with a gastric mucin phenotype, and these patterns have been evaluated together with differentiation-associated markers such as CDX2 [8]. Thus, alterations in mucin expression may reflect changes in epithelial phenotype as well as changes in mucus production.

TWEAK (TNFSF12) and its receptor Fn14 (TNFRSF12A) constitute a signaling pathway involved in tissue injury and inflammatory responses. Activation of this pathway has been reported in experimental models of intestinal inflammation and in tissues from patients with IBD [9,10,11,12]. Previous studies, including our own, have implicated TWEAK/Fn14 signaling in intestinal epithelial injury and cytokine-mediated mucosal damage, including IL-13-induced injury [13]. These findings suggest that TWEAK/Fn14 signaling may participate in epithelial responses under inflammatory conditions. However, its epithelial-intrinsic effects on goblet cell-associated mucin responses remain poorly understood.

Mouse small intestinal epithelial organoids provide an experimental system positioned between conventional epithelial cell culture and in vivo animal models. Unlike transformed epithelial cell lines, organoids retain epithelial polarity, three-dimensional organization, and differentiated epithelial lineages, including goblet cells. In contrast to in vivo models, they allow epithelial-intrinsic responses to be evaluated independently of immune cells, stromal components, and microbiota. Therefore, this model is useful for examining how inflammatory signaling affects goblet cell-associated mucin responses within the epithelial compartment.

In this study, we used mouse small intestinal epithelial organoids to investigate epithelial responses to TWEAK/Fn14-related inflammatory signaling, focusing on goblet cell-associated mucin expression and mucin composition. This study provides an organoid-based approach to examine how TWEAK/Fn14-associated inflammatory signaling affects goblet cell-related mucin responses in the epithelial compartment.

## 2. Materials and Methods

### 2.1. Animals

Six-week-old male BALB/cAJcl mice were purchased from CLEA Japan, Inc. (Tokyo, Japan) and maintained under standard conditions until they were used at 7 weeks of age for the establishment of mouse small intestinal epithelial organoids. Male mice were selected to minimize potential experimental variability associated with estrous cycle-related hormonal fluctuations during organoid establishment and subsequent analyses. No pharmacological treatment, dietary intervention, surgical procedure, or disease-inducing manipulation was performed before tissue collection. Until use, mice were maintained under specific pathogen-free conditions at the School of Allied Health Sciences, Kitasato University, under controlled environmental conditions with a temperature of 23 ± 3 °C and a 12 h light/dark cycle. Mice were housed in groups of three per cage with standard bedding and were provided ad libitum access to water and a standard laboratory diet (CE-2; CLEA Japan, Inc., Tokyo, Japan).

Animals were monitored daily for general health, and no abnormal clinical signs were observed before sample collection. At the experimental endpoint, mice were euthanized by cervical dislocation, and the small intestine was immediately collected for crypt isolation and organoid culture. All animal procedures were performed by trained personnel in accordance with institutional guidelines and the ARRIVE guidelines, and all efforts were made to minimize animal suffering. This study did not involve human participants; therefore, informed consent was not applicable.

### 2.2. Generation and Culture of Mouse Small Intestinal Epithelial Organoids

Mouse small intestinal epithelial organoids were generated with minor modifications to previously reported methods [14]. Approximately 20 cm of the proximal small intestine was collected from each of four 7-week-old male BALB/cAJcl donor mice per experimental group. Small intestinal organoids were independently established from each donor mouse. Small intestinal tissues from individual donor mice were processed separately throughout crypt isolation and organoid establishment, and crypt preparations obtained from different mice were not pooled. Organoid cultures derived from each mouse were maintained separately throughout the experiments. The luminal contents were removed by washing with ice-cold D-PBS (−) (Fujifilm Wako Pure Chemical Co., Ltd., Osaka, Japan; 045-29795). The tissue was cut into approximately 5 mm pieces and repeatedly washed with ice-cold D-PBS (−) until the supernatant became clear.

To isolate intestinal crypts, tissue fragments were incubated in D-PBS (−) containing 25 mM EDTA (Nacalai Tesque, Kyoto, Japan; 06894-14) at 37 °C for 10 min. After incubation, the tube was vigorously shaken to release crypts. The supernatant containing crypts was filtered through a 70 μm cell strainer and centrifuged at 200× *g* for 3 min at 4 °C. The crypt pellet was resuspended in basal medium, and the number of crypts was counted under a microscope.

Basal medium consisted of Advanced DMEM/F-12 (Gibco, Thermo Fisher Scientific, Waltham, MA, USA; 12634-010) supplemented with 1× penicillin–streptomycin (Fujifilm Wako Pure Chemical Co., Ltd.; 168-23191), 1× GlutaMAX (Gibco; 35050-061), and 10 mM HEPES (Sigma-Aldrich, St. Louis, MO, USA; H0887). Complete medium was prepared by supplementing basal medium with 50 ng/mL EGF (Gibco; PMG8041), 100 ng/mL Noggin (PeproTech, Cranbury, NJ, USA; 250-38-20UG), 500 ng/mL R-spondin 1 (Qkine, Cambridge, UK; Qk006-0100), 1 mM N-acetyl-L-cysteine (Fujifilm Wako Pure Chemical Co., Ltd.; 013-05133), 1× B-27 supplement (Gibco; 17504-044), and 1× N-2 supplement (Gibco; 17502-048). For initial culture after seeding, preparation medium was prepared by adding 10 μM Y-27632 (Nacalai Tesque; 08945-71) and 100 μg/mL Primocin (InvivoGen, San Diego, CA, USA; ant-pm-1) to the complete medium.

After washing with basal medium, isolated crypts were suspended in Matrigel (Corning, Corning, NY, USA; 354230) at a density of 500 crypts per well. The suspension was seeded as 25 μL domes in 48-well plates. After polymerization of Matrigel at 37 °C, 250 μL of preparation medium was gently added to each well to initiate organoid culture. The medium was changed every 3–4 days. One week after seeding, organoids were maintained in complete medium without Y-27632. The resulting three-dimensional epithelial structures were used as mouse small intestinal epithelial organoids for subsequent analyses.

### 2.3. Treatment of Mouse Small Intestinal Epithelial Organoids

On day 3 after the initiation of organoid culture, the culture medium was removed using a Pasteur pipette and replaced with 250 μL of fresh complete medium containing 100 μg/mL Primocin. Organoids were assigned to the following treatment groups: 100 ng/mL recombinant mouse TWEAK/TNFSF12 (HEK293-derived, Fc-tagged; MedChemExpress, Monmouth Junction, NJ, USA; catalog no. HY-P77264); 20 ng/mL recombinant mouse TNF (Fujifilm Wako Pure Chemical Co., Ltd., Osaka, Japan; catalog no. 201-13461); 100 ng/mL TWEAK combined with 10 μg/mL anti-mouse TNF neutralizing antibody (hereafter referred to as anti-TNF; Selleck Chemicals, Houston, TX, USA; catalog no. A2124); and 10 μg/mL anti-TNF alone. Control organoids were cultured in the same medium without TWEAK, TNF, or anti-TNF. The treatment concentrations were selected based on previous studies and the intended experimental endpoints. TWEAK at 100 ng/mL has been used to examine TWEAK/Fn14-mediated responses in intestinal epithelial cells [13,15], whereas TNF at 20 ng/mL has been used to induce epithelial responses in intestinal organoid or enteroid models [16,17]. The anti-TNF antibody concentration of 10 μg/mL was selected based on previous in vitro studies in which a neutralizing anti-mouse TNF antibody of the same clone (XT3.11) was used at 10 μg/mL to inhibit TNF activity [18]. A 3-day treatment period was chosen to permit assessment of both gene expression and morphological, goblet cell-associated, and mucin-related changes. After 3 days of treatment, control and treated organoids were subjected to gene expression, histological, immunostaining, and mucus staining analyses.

### 2.4. Gene Expression Analysis by Quantitative Real-Time PCR

Organoids were recovered from Matrigel using Cell Recovery Solution (Corning, Corning, NY, USA; 354253). Total RNA was extracted from organoids using TRIzol Reagent (Invitrogen, Thermo Fisher Scientific, Waltham, MA, USA; 15596026) according to the manufacturer’s instructions. Complementary DNA (cDNA) was synthesized from total RNA by reverse transcription using the PrimeScript RT Reagent Kit (Takara Bio Inc., Shiga, Japan; RR037A). Real-time PCR was performed using SYBR Green PCR Master Mix (Applied Biosystems, Thermo Fisher Scientific, Waltham, MA, USA; 4309155). Amplification was carried out using the ABI 7500 Real-Time PCR System (Applied Biosystems, Foster City, CA, USA). The thermal cycling conditions were as follows: initial denaturation at 95 °C for 10 min, followed by 40 cycles of denaturation at 95 °C for 15 s and annealing/extension at 60 °C for 1 min. Relative gene expression levels were calculated using the comparative Ct (2^−ΔΔCt^) method and normalized to *Gapdh* as the internal control. The primer sequences used in this study were as follows: *E-cadherin/Cdh1*, forward 5′-CTACAGCATCACCGGCCAA-3′ and reverse 5′-ACACGGCATGAGAATAGAGGATG-3′; *Lgr5*, forward 5′-TCTGCTTCCTAGAAGAGTTACGTC-3′ and reverse 5′-ATGTGGTTGGCATCTAGGCG-3′; *Ki-67/Mki67*, forward 5′-CCTGTGAGGCTGAGACATGG-3′ and reverse 5′-CGCCTTGATGGTTCCTTTCC-3′; *Muc2*, forward 5′-GTCACGATGACCACTGAGCA-3′ and reverse 5′-AGGTCTCTCGATCACCACCA-3′; *Muc5ac*, forward 5′-CCACTTTCTCCTTCTCCACACC-3′ and reverse 5′-GGTTGTCGATGCAGCCTTGCTT-3′; *Muc6*, forward 5′-GGAACTAACAGTCTGGACCACC-3′ and reverse 5′-CTTCGGTATGGATGTAGGAGGC-3′; *Tnf,* forward 5′-GGTGCCTATGTCTCAGCCTCTT-3′ and reverse 5′-GCCATAGAACTGATGAGAGGGA-3′; Ifng, forward 5′-CAGCAACAGCAAGGCGAAAAAGG-3′ and reverse 5′-TTTCCGCTTCCTGAGGCTGGAT-3′; Il6, forward 5′-TACCACTTCACAAGTCGGAGGC-3′ and reverse 5′-CTGCAAGTGCATCATCGTTGTTC-3′; Il1b, forward 5′-TGGACCTTCCAGGATGAGGACA-3′ and reverse 5′-GTTCATCTCGGAGCCTGTAGTG-3′; Klf4, forward 5′- CTATGCAGGCTGTGGCAAAACC-3′ and reverse 5′- TTGCGGTAGTGCCTGGTCAGTT-3′; Spdef, forward 5′- CACGTTGGATGAGCACTCGCTA-3′ and reverse 5′- AGCCACTTCTGCACGTTACCAG-3′; and *Gapdh*, forward 5′-AACTTTGGCATTGTTGTGGAAGG-3′ and reverse 5′-ACACATTGGGGGTAGGAACA-3′.

### 2.5. Histological, Immunohistochemical, and Mucus Staining Analysis

Small intestinal tissues and organoids were processed for histological, immunohistochemical, immunofluorescence, and mucus staining analyses, as appropriate. Small intestinal tissues were used mainly to examine the localization of Fn14 in the epithelial compartment, whereas organoids were used to evaluate changes in goblet cell-associated markers and mucus-related staining after each treatment.

For paraffin sections, samples were fixed in freshly prepared 4% paraformaldehyde in phosphate-buffered saline (PBS) for 30 min at room temperature and embedded in paraffin [19]. Paraffin-embedded samples were sectioned at 4 μm thickness, deparaffinized in xylene, and rehydrated through a graded ethanol series. For frozen sections, samples were embedded in O.C.T. Compound, frozen, sectioned at 7 μm thickness, and fixed with 4% paraformaldehyde before staining.

For immunostaining, heat-induced antigen retrieval was performed in 10 mM citrate buffer, pH 6.0, at 95 °C for 15 min, followed by gradual cooling to room temperature. Sections were permeabilized with 0.5% Triton X-100 in PBS for 10 min at room temperature when required. For horseradish peroxidase (HRP)/3,3′-diaminobenzidine (DAB)-based detection, endogenous peroxidase activity was blocked by incubation with 3% H_2_O_2_ in PBS for 10 min at room temperature. After washing with PBS, sections were blocked with Protein Block (Agilent Technologies, Santa Clara, CA, USA; catalog no. X0909) for 30 min at room temperature.

The following primary antibodies were used: rabbit anti-mouse Fn14 antibody (Cell Signaling Technology, Danvers, MA, USA; catalog no. 4403S; dilution 1:200), goat anti-mouse Fn14 antibody (R&D Systems, Minneapolis, MN, USA; catalog no. AF1610; dilution 1:20), goat anti-mouse E-cadherin antibody (R&D Systems; catalog no. AF748; dilution 1:200), rabbit anti-mouse MUC2 antibody (Santa Cruz Biotechnology, Dallas, TX, USA; catalog no. sc-515032; dilution 1:400), and PGM34 antibody, an in-house antibody recognizing sulfomucin, as previously described [20,21,22] (dilution 1:40). Primary antibodies were diluted in Antibody Diluent with Background-Reducing Components (Agilent Technologies, Santa Clara, CA, USA; catalog no. S3022) and incubated overnight at 4 °C.

For fluorescence detection, sections were washed with PBS and incubated with appropriate secondary antibodies for 30 min at room temperature. The secondary antibodies used were anti-rabbit IgG conjugated to Alexa Fluor 488 (Cell Signaling Technology; catalog no. 4412S; dilution 1:500) and anti-goat IgG conjugated to Alexa Fluor 594 (Abcam, Cambridge, UK; catalog no. ab150140; dilution 1:1000). Nuclei were counterstained with DAPI (Dojindo Laboratories, Kumamoto, Japan), and sections were mounted using Fluorescence Mounting Medium (Agilent Technologies, Santa Clara, CA, USA).

For HRP/DAB-based chromogenic detection, sections were incubated with EnVision+ System-HRP-Labeled Polymer Anti-Rabbit (Agilent Technologies, Santa Clara, CA, USA; catalog no. K4003) according to the manufacturer’s instructions. For mouse primary antibodies, the M.O.M. ImmPRESS Polymer Kit (Vector Laboratories, Newark, CA, USA; catalog no. MP-2400) was used according to the manufacturer’s protocol. Color development was performed using ImmPACT DAB Substrate Kit (Vector Laboratories; catalog no. SK-4105), followed by nuclear counterstaining with hematoxylin. Sections were then dehydrated through a graded ethanol series, cleared in xylene, and mounted.

For mucus staining of organoids, periodic acid–Schiff (PAS) staining was performed using a PAS staining kit (Muto Pure Chemicals Co., Ltd., Tokyo, Japan; catalog no. 15792) according to the manufacturer’s instructions. Alcian blue–PAS (AB-PAS) staining was performed by combining the PAS staining kit with Alcian blue staining solution (Muto Pure Chemicals Co., Ltd.; catalog no. 40852) to evaluate mucin composition.

Fluorescence images were acquired using a Nikon C2si laser scanning confocal microscope (Nikon Corporation, Tokyo, Japan). Bright-field images were obtained using an Olympus BX53 light microscope (Olympus Corporation, Tokyo, Japan). Image processing and quantitative analyses were performed using ImageJ software 1.54g (National Institutes of Health, Bethesda, MD, USA). The total area of each organoid was measured. The number of MUC2-positive cells was counted and normalized to a defined organoid area. For PAS, AB-PAS, and PGM34 staining, the positively stained area was measured and expressed as a percentage of the total organoid area.

### 2.6. Statistical Analysis

Data are presented as the mean ± standard error of the mean (SEM). For gene-expression analyses, the organoid culture sample was used as the statistical unit, and the *n* values indicate the number of culture samples analyzed. For histological and imaging analyses, including measurements of organoid area, MUC2-positive cells, PAS-positive area, AB-PAS staining, and PGM34-positive area, individual organoids were quantified and used in the statistical comparisons.

Normality of the data distribution was assessed using the Shapiro–Wilk test before parametric statistical testing. Statistical comparisons between two groups were performed using an unpaired Welch’s *t*-test, whereas comparisons among multiple groups were performed using one-way ANOVA followed by Tukey’s multiple comparisons test, as appropriate. All statistical analyses were conducted using GraphPad Prism version 10.6.1 (GraphPad Software, Inc., La Jolla, CA, USA). All tests were two-tailed, and *p*-values < 0.05 were considered statistically significant.

### 2.7. Use of Generative AI Tools

Generative AI tools were used only for language editing and improving readability. The authors reviewed and approved all final content and take full responsibility for the manuscript.

## 3. Results

### 3.1. Fn14 Is Expressed in Goblet Cells of the Small Intestinal Epithelium

**Figure 1:**
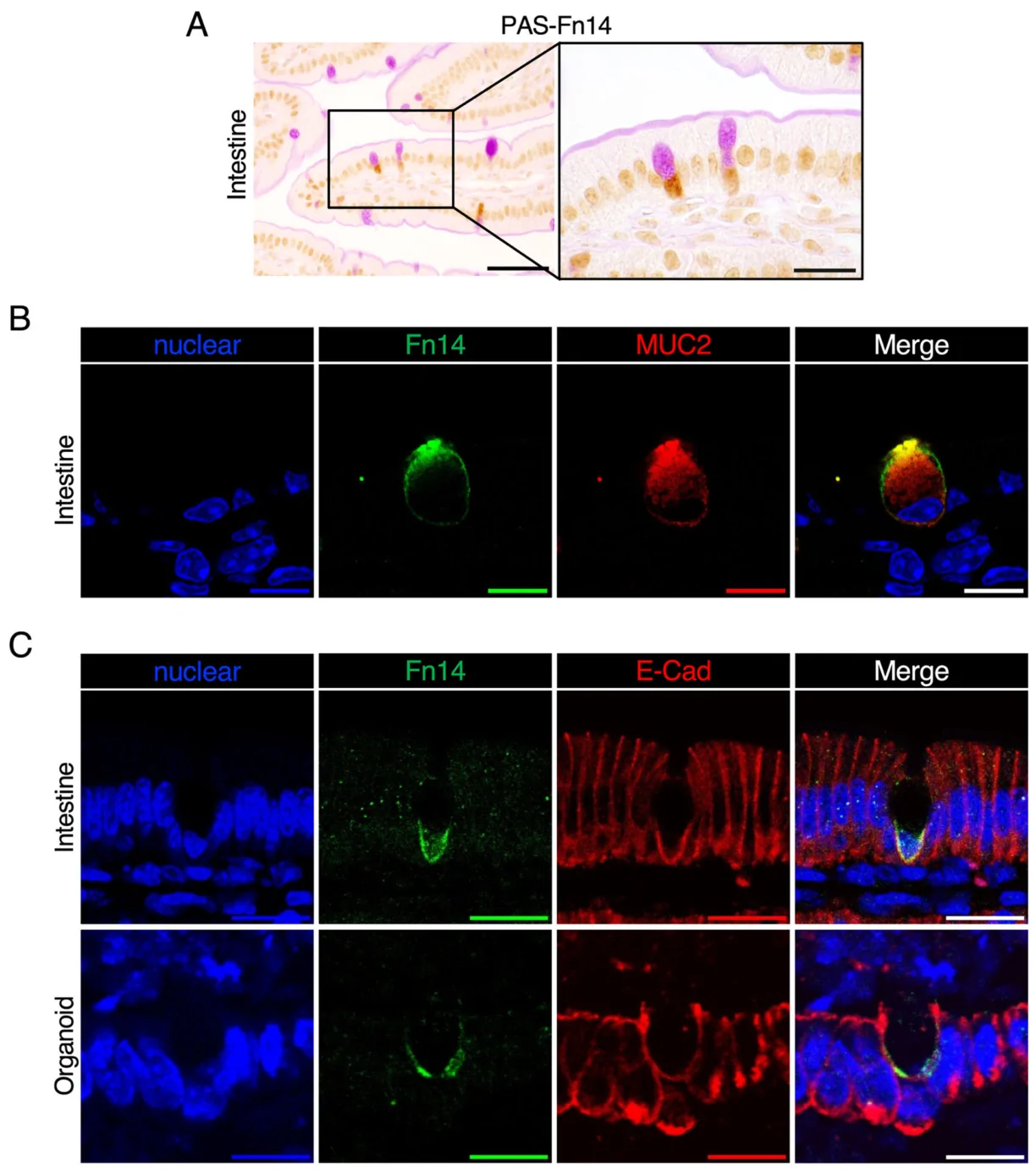
Localization of Fn14 in goblet cells of the mouse small intestine and small intestinal epithelial organoids. (**A**) Representative PAS-DAB-stained images of the mouse small intestine showing mucin (pink) and Fn14 (brown). Scale bars = 50 μm (left) and 20 μm (right). (**B**) Representative immunofluorescence images of the mouse small intestine co-stained for Fn14 (green) and MUC2 (red). Scale bar = 20 μm. (**C**) Representative immunofluorescence images of the mouse small intestine and small intestinal epithelial organoids co-stained for Fn14 (green) and E-cadherin (red). Scale bars = 20 μm. For each specimen, 10 fields were examined. Representative images showing staining patterns consistently observed across the examined fields are presented.

Our research group previously demonstrated that Fn14 is expressed in the colonic epithelium and that the TWEAK/Fn14 pathway is involved in intestinal epithelial injury and inflammation [12]. Because the small intestinal epithelium shares structural similarities with the colonic epithelium, Fn14 may also be expressed and function in the small intestine. Therefore, we examined the localization of Fn14 in mouse small intestinal tissue using PAS-DAB staining. Fn14 expression was detected in mucus-producing cells in the small intestinal epithelium (Figure 1A).

Goblet cells are the major mucus-producing cells in the small intestine and are characterized by the production of Muc2-positive mucins [23]. Immunofluorescence analysis of Fn14 and Muc2 revealed that Fn14 was expressed in Muc2-positive cells (Figure 1B). Fn14 staining was preferentially observed toward the mucin-facing region of MUC2-positive cells. To determine whether Fn14 was expressed on the cell membrane, co-staining with E-cadherin was performed. Fn14 co-localized with the cell membrane marker E-cadherin in both small intestinal tissue and organoids (Figure 1C). Notably, Fn14 staining was preferentially observed along the inner edge of MUC2-positive cells adjacent to mucin rather than along the outer edge.

### 3.2. TWEAK Treatment Increases TNF mRNA Expression and Reduces Muc2 mRNA Expression in Small Intestinal Epithelial Organoids

**Figure 2:**
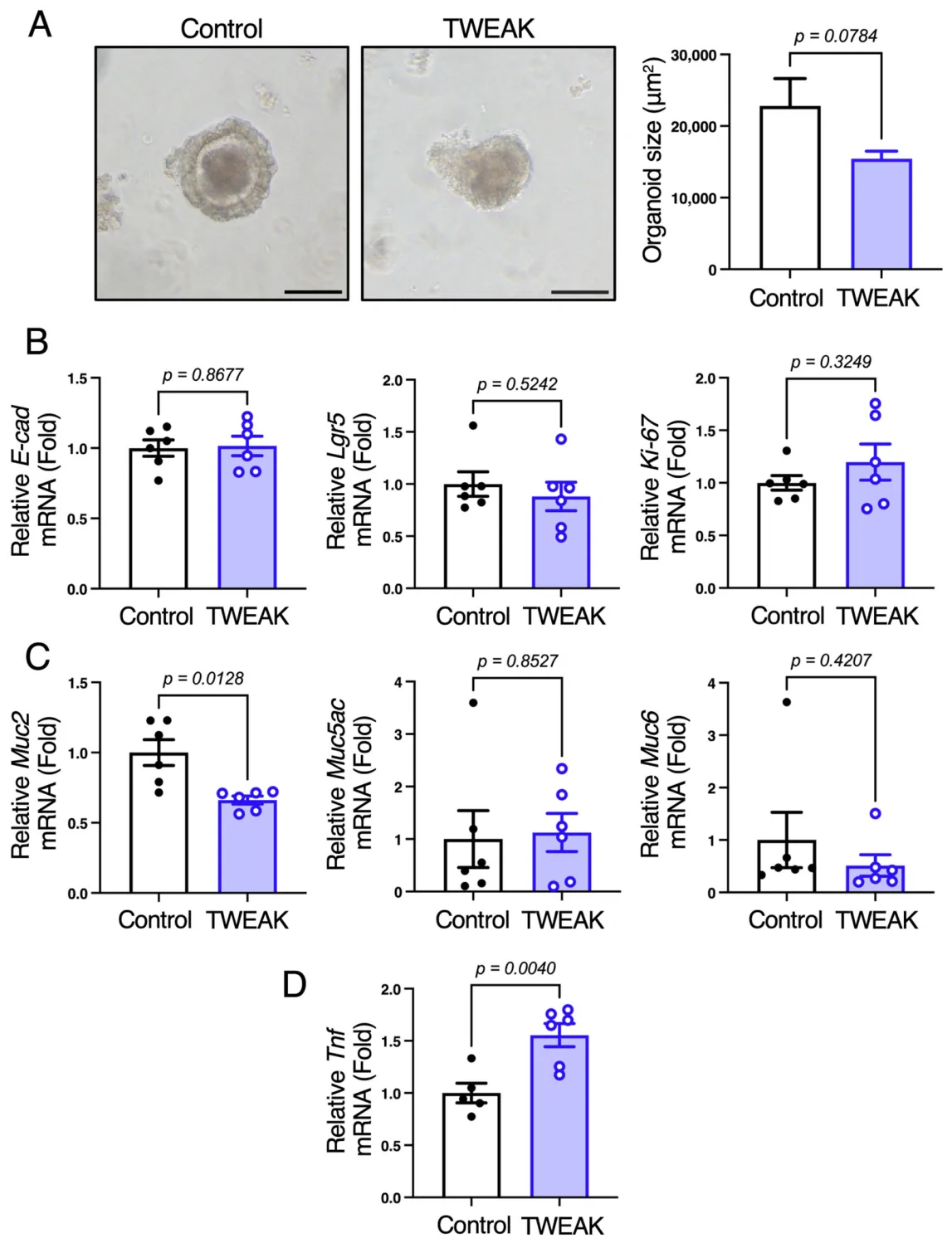
Effects of TWEAK treatment on organoid growth and gene expression in small intestinal epithelial organoids. (**A**) Representative bright-field images of control and TWEAK-treated organoids during culture. Scale bar = 100 μm. The bar graph shows the quantified organoid area (*n* = 19 organoids per group). (**B**) Relative expression levels of *E-cadherin* (*E-cad*), *Lgr5*, and *Ki-67* measured by quantitative real-time PCR (*n* = 6). (**C**) Relative expression levels of the mucin-related genes *Muc2*, *Muc5ac*, and *Muc6* measured by quantitative real-time PCR (*n* = 6). (**D**) Relative Tnf mRNA expression measured by quantitative real-time PCR (*n* = 6). Organoid cultures were derived from four donor mice per group. A total of six culture samples per group were analyzed, with one or two culture samples obtained from each donor mouse. These culture samples were analyzed in two independent experiments. Data are presented as the mean ± standard error of the mean (SEM). Statistical comparisons were performed using an unpaired Welch’s *t*-test. Exact *p*-values are shown in the graphs, and *p* < 0.05 was considered statistically significant.

To investigate the function of Fn14 expressed in goblet cells, mouse small intestinal epithelial organoids were treated with TWEAK, the ligand for Fn14, and changes in gene expression were analyzed. TWEAK treatment tended to reduce organoid size, although the difference was not statistically significant (Figure 2A). Analysis of intestinal epithelial markers showed that TWEAK treatment did not significantly affect the expression of *E-cadherin* (*E-cad*), which is essential for intercellular adhesion, or *Lgr5* and *Ki-67*, which are associated with stem cell function and cell proliferation, respectively (Figure 2B).

Because Fn14 was expressed in goblet cells, we hypothesized that the TWEAK/Fn14 pathway might regulate mucin-related functions. TWEAK treatment reduced *Muc2* mRNA expression to approximately 0.66-fold that of the control group, whereas *Muc5ac* and *Muc6* mRNA expression remained unchanged (Figure 2C). To further assess whether the reduction in *Muc2* mRNA expression was accompanied by changes in goblet cell differentiation-associated genes, we examined *Klf4* and *Spdef* mRNA expression. TWEAK treatment did not significantly alter either *Klf4* or *Spdef* mRNA expression compared with the control group (Figure S1). These findings indicate that the reduction in *Muc2* mRNA following TWEAK treatment was not accompanied by detectable changes in the goblet cell differentiation-associated genes *Klf4* and *Spdef* under the conditions examined. Since TWEAK has also been reported to induce inflammatory responses [12,24], we examined the expression of inflammatory cytokines. TWEAK treatment increased *Tnf* mRNA expression by approximately 1.56-fold compared with the control group (Figure 2D), whereas specific amplification of Ifng, Il6, and Il1b was not detected under the experimental conditions used (Figure S2). These findings show that TWEAK treatment was associated with increased *Tnf* mRNA expression and reduced *Muc2* mRNA expression, suggesting that TNF may contribute to some of the epithelial responses associated with TWEAK treatment.

### 3.3. TWEAK Treatment Alters Goblet Cell-Associated Features and Mucin Composition in Small Intestinal Epithelial Organoids

**Figure 3:**
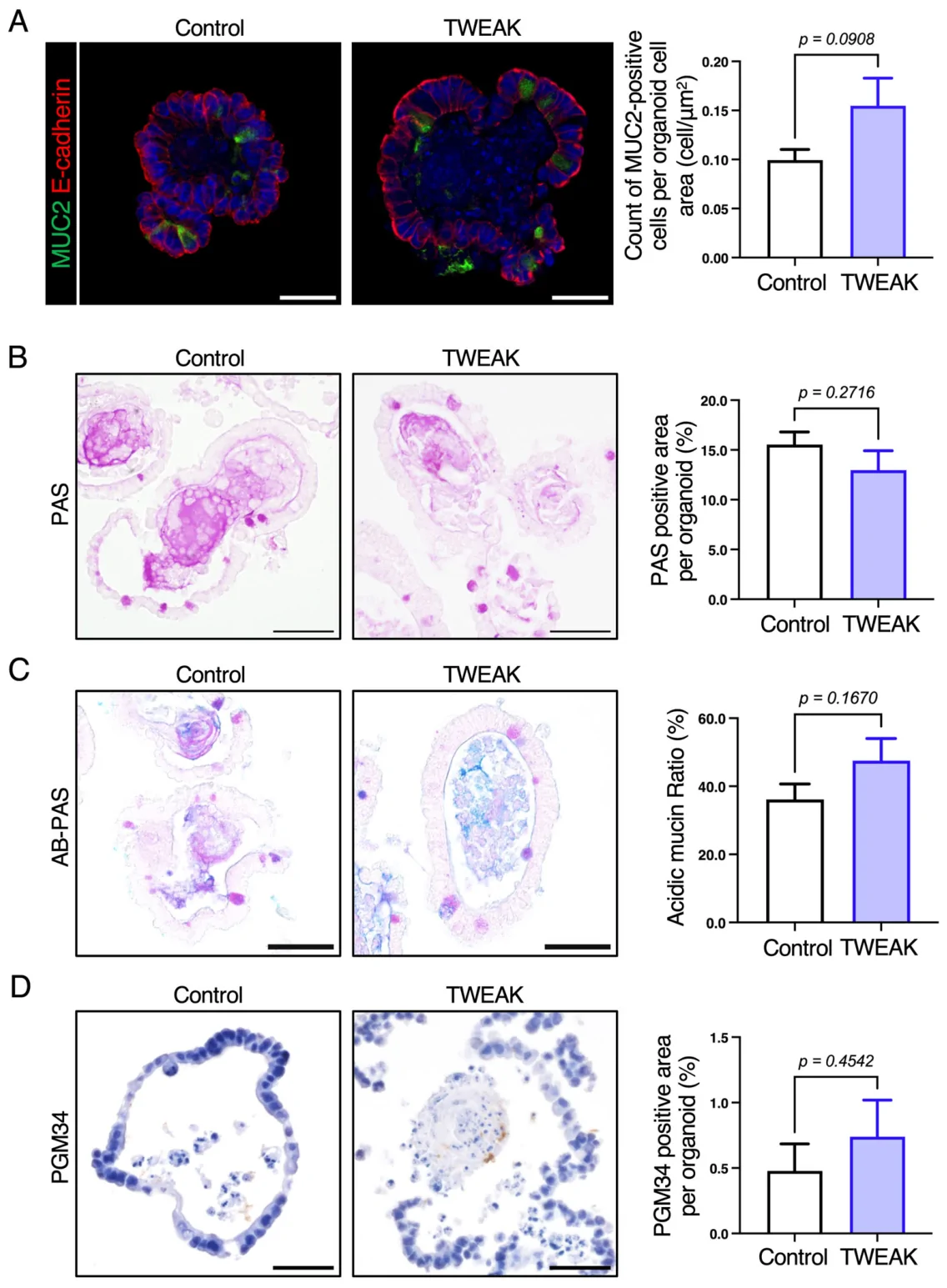
Effects of TWEAK treatment on goblet cell-associated features and mucin composition in small intestinal epithelial organoids. (**A**) Representative immunofluorescence images of control and TWEAK-treated organoids stained for E-cadherin (red) and MUC2 (green). Scale bar = 30 μm. The bar graph shows the number of MUC2-positive cells per organoid (*n* = 10–29 organoids per group). (**B**) Representative PAS-stained images of control and TWEAK-treated organoids. Scale bar = 40 μm. The bar graph shows the PAS-positive area per organoid (*n* = 29–77 organoids per group). (**C**) Representative AB-PAS-stained images of control and TWEAK-treated organoids. Scale bar = 30 μm. The bar graph shows the percentages of PAS-positive and AB-positive areas relative to the total stained area per organoid (*n* = 10–19 organoids per group). (**D**) Representative images of control and TWEAK-treated organoids stained with the anti-PGM34 antibody and visualized using DAB (brown). Scale bar = 40 μm. The bar graph shows the PGM34-positive area per organoid (*n* = 9–18 organoids per group). Organoid cultures were derived from four donor mice per group. A total of six culture samples per group were analyzed, with one or two culture samples obtained from each donor mouse. These culture samples were analyzed in two independent experiments. The *n* values shown for histological analyses indicate the number of individual organoids analyzed. Data are presented as the mean ± standard error of the mean (SEM). Statistical comparisons were performed using an unpaired Welch’s *t*-test. Exact *p*-values are shown in the graphs, and *p* < 0.05 was considered statistically significant.

Because TWEAK treatment reduced *Muc2* mRNA expression, we next examined whether this change was accompanied by alterations in MUC2-positive cell number and mucin-associated staining parameters. The number of MUC2-positive cells showed a non-significant tendency to increase to approximately 1.56-fold that of the control group (Figure 3A). In addition, E-cadherin expression was enhanced along the basement membrane side of the organoids (Figure 3A).

PAS staining was then performed to assess overall mucin content. TWEAK treatment did not significantly alter the PAS-positive area (Figure 3B). Analysis of mucin composition by AB-PAS staining revealed no substantial changes following TWEAK treatment (Figure 3C). PGM34 staining, which detects sulfomucin, also showed a non-significant tendency to increase to approximately 1.55-fold that of the control group following TWEAK treatment (Figure 3D).

Taken together, TWEAK treatment did not significantly alter the PAS-positive mucin area or AB-PAS-defined mucin composition, whereas both MUC2-positive cell number and PGM34 staining showed non-significant increasing trends.

### 3.4. TNF Treatment Reduces the Expression of Proliferation-Associated Markers, E-Cadherin, and Muc2 in Small Intestinal Epithelial Organoids

TWEAK treatment increased *Tnf* mRNA expression in mouse small intestinal epithelial organoids, suggesting that TNF may contribute to the gene-expression changes associated with TWEAK treatment. To examine this possibility, mouse small intestinal epithelial organoids were treated with TNF, and its effects were analyzed. TNF has been reported to induce inflammation and epithelial cell loss in the intestinal epithelium and to impair intestinal stem cell function [25,26,27,28]. Therefore, TNF treatment was expected to affect organoid growth and epithelial homeostasis.

**Figure 4:**
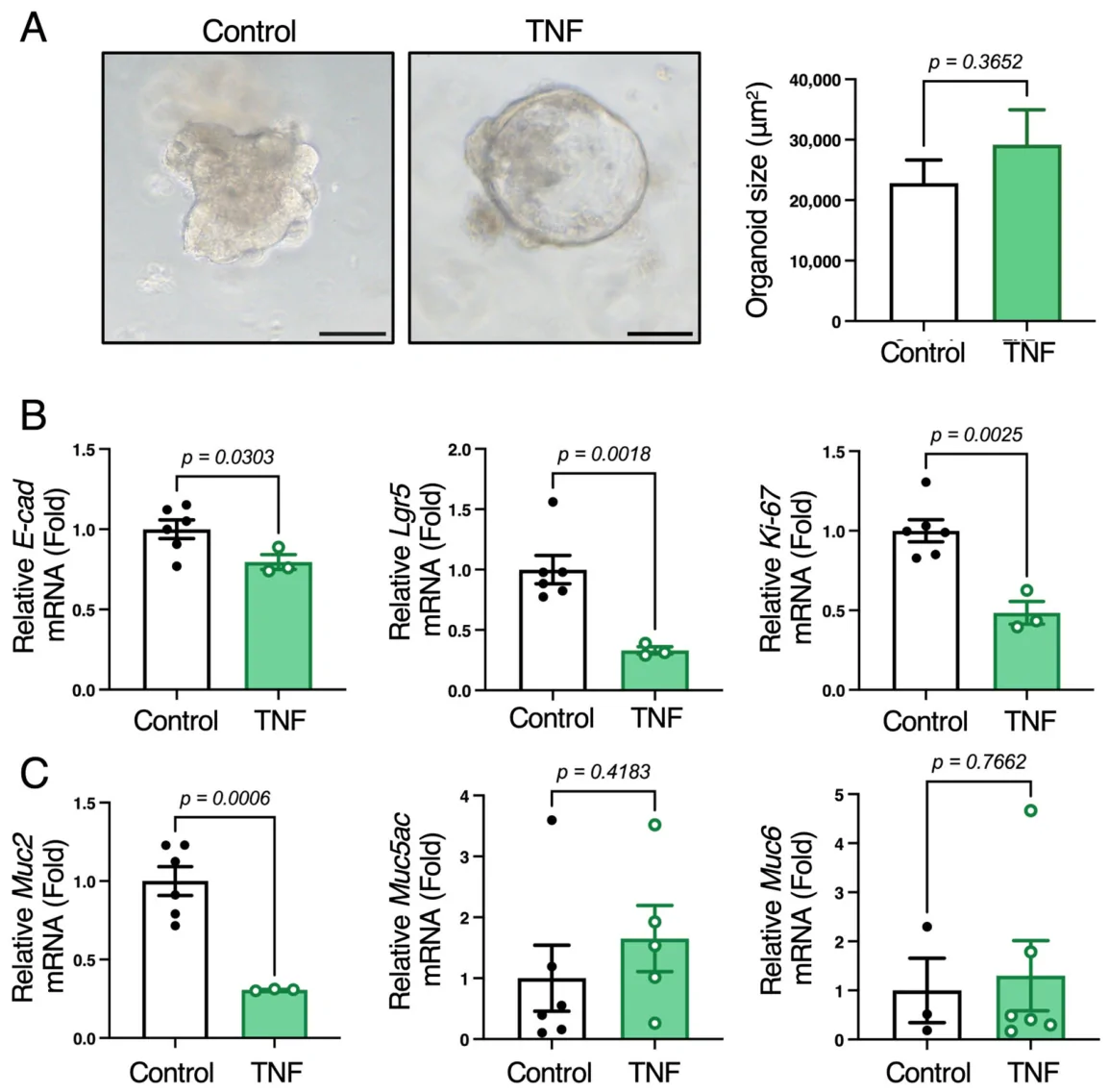
Effects of TNF treatment on organoid growth and gene expression in small intestinal epithelial organoids. (**A**) Representative bright-field images of control and TNF-treated organoids during culture. Scale bar = 100 μm. The bar graph shows the quantified organoid area (*n* = 17–19 organoids per group). (**B**) Relative expression levels of *E-cadherin*, *Lgr5*, and *Ki-67* measured by quantitative real-time PCR (*n* = 3–6). (**C**) Relative expression levels of mucin-related genes measured by quantitative real-time PCR (*n* = 3–6). Organoid cultures were derived from four donor mice per group. A total of six culture samples per group were analyzed, with one or two culture samples obtained from each donor mouse. These culture samples were analyzed in two independent experiments. The *n* values indicate the number of organoid culture samples analyzed for each gene. Data are presented as the mean ± standard error of the mean (SEM). Statistical comparisons were performed using an unpaired Welch’s *t*-test. Exact *p*-values are shown in the graphs, and *p* < 0.05 was considered statistically significant.

Analysis of organoid size during culture showed a tendency toward an increase following TNF treatment, although the difference was not statistically significant (Figure 4A). Gene expression analysis showed that TNF treatment significantly reduced *E-cadherin*, *Lgr5*, and *Ki-67* mRNA expression to approximately 0.80-fold, 0.33-fold, and 0.48-fold that of the control group, respectively (Figure 4B). These findings suggest that TNF may impair intercellular adhesion, stem cell function, and proliferative activity.

Analysis of mucin-related genes showed that TNF treatment significantly reduced *Muc2* mRNA expression to approximately 0.31-fold that of the control group, consistent with the effect observed following TWEAK treatment (Figure 4C). TNF treatment also increased *Muc5ac* expression to approximately 1.65-fold that of the control group, although the difference was not statistically significant.

Taken together, these findings suggest that TNF may contribute to the reduction in *Muc2* mRNA expression associated with TWEAK treatment and may also disrupt epithelial homeostasis by suppressing genes associated with intercellular adhesion, stem cell function, and cell proliferation.

### 3.5. TNF Treatment Reduces the Number of MUC2-Positive Cells and Alters Mucin Content and Composition in Small Intestinal Epithelial Organoids

Because TNF treatment altered mucin-related gene expression, we next examined MUC2-positive cell number, overall mucin content, and mucin composition. Histological and immunostaining analyses were performed for these assessments.

**Figure 5:**
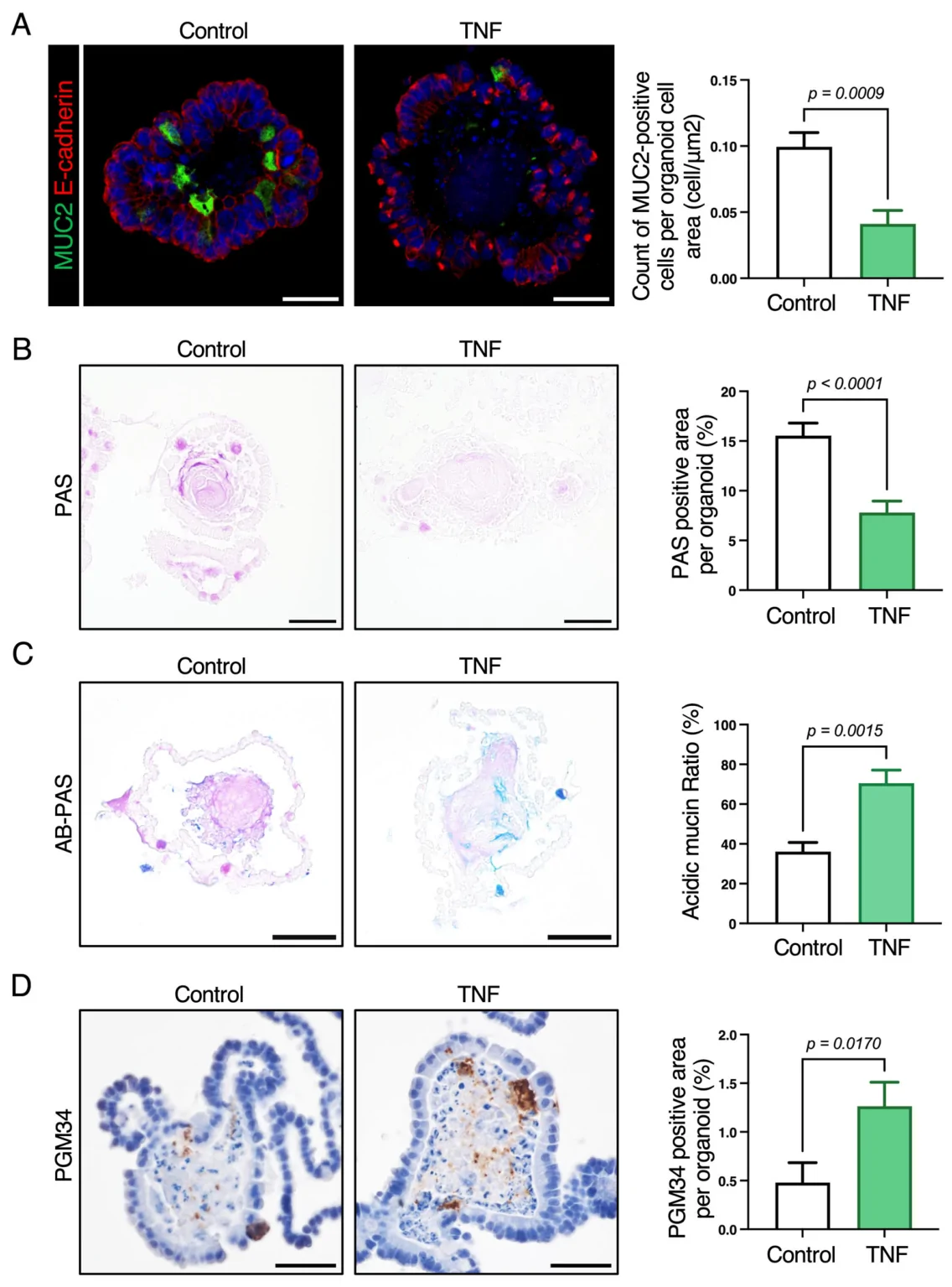
Effects of TNF treatment on goblet cell-associated features and mucin composition in small intestinal epithelial organoids. (**A**) Representative immunofluorescence images of control and TNF-treated organoids stained for E-cadherin (red) and MUC2 (green). Scale bar = 20 μm. The bar graph shows the number of MUC2-positive cells per organoid (*n* = 6–29 organoids per group). (**B**) Representative PAS-stained images of control and TNF-treated organoids. Scale bar = 40 μm. The bar graph shows the PAS-positive area per organoid (*n* = 29–77 organoids per group). (**C**) Representative AB-PAS-stained images of control and TNF-treated organoids. Scale bar = 30 μm. The bar graph shows the percentages of PAS-positive and AB-positive areas relative to the total stained area per organoid (*n* = 18–19 organoids per group). (**D**) Representative images of control and TNF-treated organoids stained with the anti-PGM34 antibody and visualized using DAB (brown). Scale bar = 40 μm. The bar graph shows the PGM34-positive area per organoid (*n* = 18–23 organoids per group). Organoid cultures were derived from four donor mice per group. A total of six culture samples per group were analyzed, with one or two culture samples obtained from each donor mouse. These culture samples were analyzed in two independent experiments. The *n* values shown for histological analyses indicate the number of individual organoids analyzed. Data are presented as the mean ± standard error of the mean (SEM). Statistical comparisons were performed using an unpaired Welch’s *t*-test. Exact *p*-values are shown in the graphs, and *p* < 0.05 was considered statistically significant.

The number of MUC2-positive cells decreased significantly to approximately 0.41-fold that of the control group following TNF treatment (Figure 5A). In addition, E-cadherin staining was enhanced along the basement membrane side of the organoids but reduced on the luminal side (Figure 5A).

PAS staining showed that the PAS-positive area decreased significantly to approximately 0.50-fold that of the control group following TNF treatment, indicating a reduction in overall mucin content (Figure 5B). In contrast, AB-PAS staining revealed that the proportion of AB-positive acidic mucin increased significantly to approximately 1.95-fold that of the control group (Figure 5C). These findings indicate that TNF treatment reduced overall mucin content while shifting the mucin composition toward a higher proportion of acidic mucin. In PGM34 staining to detect sulfomucin, PGM34 staining increased significantly to approximately 2.64-fold that of the control group following TNF treatment (Figure 5D).

Taken together, these findings indicate that TNF treatment reduces the number of MUC2-positive cells and overall mucin content while increasing the proportion of acidic mucin in small intestinal epithelial organoids.

### 3.6. TNF Inhibition Modifies TWEAK-Associated Changes in E-Cadherin and Muc2 mRNA Expression in Small Intestinal Epithelial Organoids

TWEAK treatment increased *TNF* mRNA expression and reduced *Muc2* mRNA expression in small intestinal epithelial organoids. In addition, direct TNF treatment also reduced *Muc2* mRNA expression. Based on these observations, we examined whether treatment with a neutralizing anti-TNF antibody modified the epithelial responses associated with TWEAK treatment. Organoids were treated with the anti-TNF antibody alone or in combination with TWEAK.

**Figure 6:**
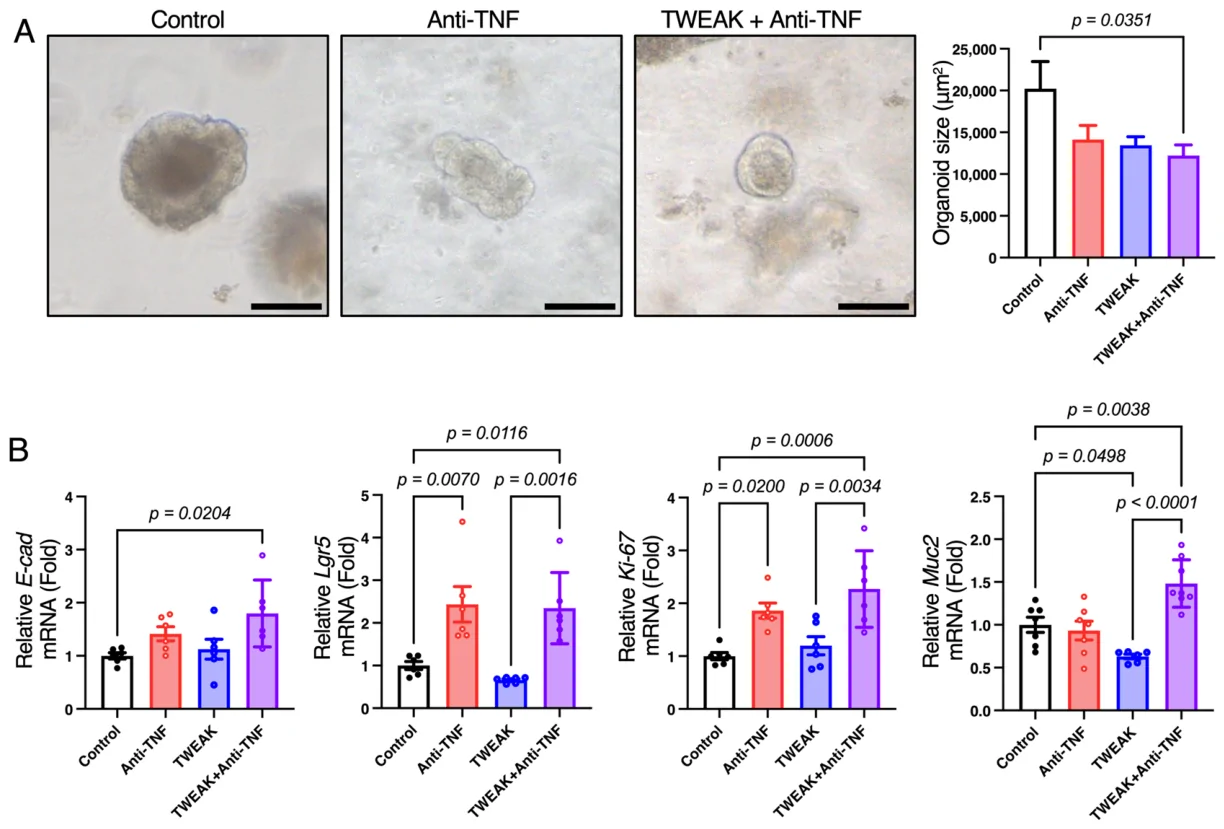
Effects of TNF inhibition on TWEAK-associated changes in organoid growth and gene expression in small intestinal epithelial organoids. (**A**) Representative bright-field images of control, TWEAK-treated, TNF inhibitor-treated (Anti-TNF), and combined TWEAK plus TNF inhibitor (Anti-TNF)-treated organoids during culture. Scale bar = 100 μm. The bar graph shows the quantified organoid area (*n* = 21–60 organoids per group). (**B**) Relative expression levels of *E-cadherin*, *Lgr5, Ki-67*, and *Muc2* measured by quantitative real-time PCR (*n* = 6–8). Organoid cultures were derived from four donor mice per group. A total of 6–8 culture samples per group were analyzed, with one or two culture samples obtained from each donor mouse. These culture samples were analyzed in two independent experiments. Data are presented as the mean ± standard error of the mean (SEM). Statistical comparisons were performed using one-way analysis of variance followed by Tukey’s multiple-comparison test. Adjusted *p*-values are shown in the graphs, and adjusted *p* < 0.05 was considered statistically significant.

Treatment with either TWEAK or the TNF inhibitor alone tended to reduce organoid size, whereas combined treatment significantly reduced organoid size to approximately 0.60-fold that of the control group (Figure 6A).

Gene expression analysis showed that *E-cad* mRNA expression was significantly increased to approximately 1.80-fold that of the control group only after combined treatment with TWEAK and the TNF inhibitor (Figure 6B). Treatment with the TNF inhibitor increased *Lgr5* and *Ki-67* mRNA expression regardless of the presence or absence of TWEAK. *Muc2* mRNA expression was not altered by treatment with the TNF inhibitor alone but was significantly increased to approximately 1.48-fold that of the control group following combined treatment with TWEAK and the TNF inhibitor (Figure 6B).

Taken together, these findings suggest that TNF inhibition may modify the response of small intestinal epithelial organoids to TWEAK, particularly with respect to E-cadherin and MUC2 expression.

### 3.7. Combined Treatment with TWEAK and a TNF Inhibitor Alters Goblet Cell-Associated Features and Mucin Composition in Small Intestinal Epithelial Organoids

**Figure 7:**
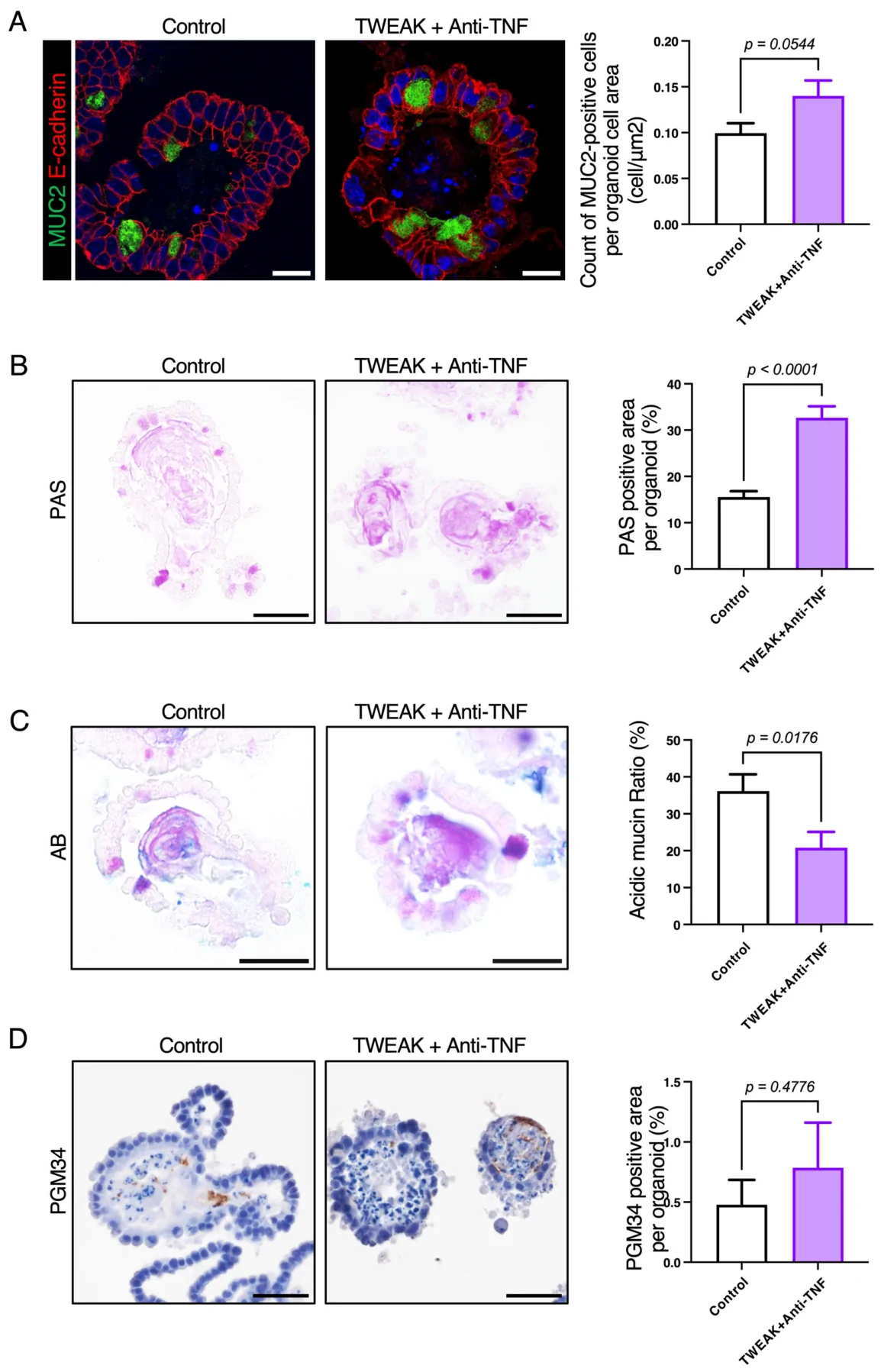
Effects of combined TWEAK and TNF inhibitor treatment on goblet cell-associated features and mucin composition in small intestinal epithelial organoids. (**A**) Representative immunofluorescence images of control and combined TWEAK plus TNF inhibitor-treated organoids stained for E-cadherin (red) and MUC2 (green). Scale bar = 50 μm. The bar graph shows the number of MUC2-positive cells per organoid (*n* = 27–52 organoids per group). (**B**) Representative PAS-stained images of control and combined treatment organoids. Scale bar = 40 μm. The bar graph shows the PAS-positive area per organoid (*n* = 32–77 organoids per group). (**C**) Representative AB-PAS-stained images of control and combined treatment organoids. Scale bar = 30 μm. The bar graph shows the percentages of PAS-positive and AB-positive areas relative to the total stained area per organoid (*n* = 19–23 organoids per group). (**D**) Representative images of control and combined treatment organoids stained with the anti-PGM34 antibody and visualized using DAB (brown). Scale bar = 40 μm. The bar graph shows the PGM34-positive area per organoid (*n* = 18–33 organoids per group). Organoid cultures were derived from four donor mice per group. A total of six culture samples per group were analyzed, with one or two culture samples obtained from each donor mouse. These culture samples were analyzed in two independent experiments. The *n* values shown for histological analyses indicate the number of individual organoids analyzed. Data are presented as the mean ± standard error of the mean (SEM). Statistical comparisons were performed using an unpaired Welch’s *t*-test. Exact *p*-values are shown in the graphs, and *p* < 0.05 was considered statistically significant.

To examine the effects of combined treatment with TWEAK and a TNF inhibitor on goblet cells and mucin properties, immunostaining and histological analyses were performed. Immunofluorescence staining for MUC2 showed that the number of MUC2-positive cells tended to increase following combined treatment, although the difference was not statistically significant (Figure 7A).

PAS staining was performed to assess overall mucin content. The PAS-positive area increased significantly following combined treatment (Figure 7B). Analysis of mucin composition by AB-PAS staining showed that the proportion of AB-positive acidic mucin decreased significantly to approximately 0.57-fold that of the control group (Figure 7C).

To further characterize acidic mucin composition, staining with PGM34, which detects sulfomucin, was performed. PGM34 staining tended to increase following combined treatment with TWEAK and the TNF inhibitor (Figure 7D).

Taken together, these findings suggest that combined treatment with TWEAK and a TNF inhibitor may affect goblet cell-associated mucin properties, particularly by decreasing the proportion of acidic mucin in small intestinal epithelial organoids.

## 4. Discussion

In this study, we used mouse small intestinal epithelial organoids to investigate the epithelial-intrinsic role of the TWEAK/Fn14 pathway. Cytokines regulate epithelial apoptosis, proliferation, and barrier function through interactions between epithelial and immune cells [29,30,31]. Consequently, epithelial-specific signaling pathways can be difficult to evaluate in vivo because of complex immune–epithelial crosstalk. Immune cells also contribute to epithelial differentiation and intestinal homeostasis [32,33,34], underscoring their physiological importance. However, an epithelial-only system is useful for examining signaling responses that arise within epithelial cells independently of immune-derived factors. Small intestinal epithelial organoids consist primarily of epithelial cells and retain key features of epithelial organization and homeostasis [14,35,36], making them suitable for this purpose. Although organoids do not fully reproduce the cellular and environmental complexity of the intestine in vivo, they provide a useful platform for identifying epithelial-intrinsic responses that can subsequently be examined in more complex experimental models.

We found that Fn14 was expressed in MUC2-positive cells in the mouse small intestinal epithelium. Fn14 staining was also observed in small intestinal epithelial organoids, where it co-localized with E-cadherin. Thus, Fn14 expression was observed in both intestinal tissue and the organoid model, although direct co-localization with MUC2 was demonstrated in intestinal tissue. Because goblet cells contribute to the mucus barrier separating luminal contents from the epithelial surface, Fn14 raises the possibility that TWEAK/Fn14 signaling may influence goblet cell responses at the luminal interface. However, whether Fn14 directly participates in sensing luminal factors or mucus-associated molecules remains to be determined.

TWEAK treatment did not significantly alter the expression of *Lgr5* or *Ki-67*, suggesting that TWEAK alone did not markedly affect stem cell-associated properties or proliferative activity under the conditions used in this study. This finding is consistent with previous reports indicating that TWEAK alone does not strongly induce apoptosis in intestinal epithelial cells [12,13,37]. Nevertheless, TWEAK treatment increased Tnf mRNA expression and reduced *Muc2* mRNA expression, indicating that it elicited measurable epithelial responses.

To our knowledge, the relationship between TWEAK/Fn14 signaling and MUC2 expression in the small intestinal epithelium has not been well characterized. Therefore, the present findings suggest a previously underexplored association between this pathway and goblet cell-associated mucin regulation. Interestingly, the number of MUC2-positive cells tended to increase despite the reduction in *Muc2* mRNA expression. To further examine whether this finding was associated with altered goblet cell differentiation, we analyzed *Klf4* and *Spdef*, transcription factors associated with intestinal goblet cell differentiation and maturation. Neither *Klf4* nor *Spdef mRNA* expression was significantly altered by TWEAK treatment. Thus, the reduction in *Muc2* mRNA following TWEAK treatment was not accompanied by detectable changes in these goblet cell differentiation-associated genes. Together with the absence of a significant reduction in MUC2-positive cell number, these findings are consistent with the possibility that TWEAK affects *Muc2* transcription within existing MUC2-positive cells rather than markedly reducing goblet cell abundance. However, because *Klf4* and *Spdef* alone do not provide a comprehensive assessment of goblet cell identity or differentiation state, and because goblet cell maturation and mucin secretion were not directly evaluated, this interpretation remains tentative.

TWEAK treatment did not significantly alter the overall PAS-positive mucin area or the relative proportions of neutral and acidic mucins detected by AB-PAS staining. In contrast, PGM34 staining showed a tendency to increase following TWEAK treatment. PGM34 recognizes a specific sulfated carbohydrate epitope, the 6-sulfated blood-group H type 2 antigen, present on mucin glycans [20]. Therefore, the increased PGM34 reactivity may reflect a change in specific sulfated mucin-associated glycan features rather than an increase in overall mucin production. Changes in PGM34-reactive sulfated mucins have also been reported in the mouse small intestine under inflammatory or infectious conditions [22], suggesting that mucin sulfation patterns can be dynamically regulated in response to environmental stimuli. However, because the increase in PGM34 staining did not reach statistical significance and mucin glycan structures were not directly analyzed in the present study, the biological significance of this finding remains uncertain. Further glycomic analyses will be required to determine whether TWEAK directly influences mucin sulfation or other aspects of mucin glycosylation.

TWEAK treatment increased *Tnf* mRNA expression, suggesting that TNF may contribute to some of the epithelial changes observed following TWEAK stimulation [12,24]. Although TWEAK and TNF treatments shared some responses, particularly reduced *Muc2* mRNA expression, their overall effects were not identical. TWEAK treatment did not significantly reduce the number of MUC2-positive cells, whereas TNF treatment markedly reduced MUC2-positive cell number and PAS-positive mucin area. These differences suggest that TNF may contribute to a subset of TWEAK-associated epithelial responses, but the present data do not support a simple linear TWEAK/Fn14–TNF pathway. TWEAK and TNF may also influence goblet cell-associated phenotypes through partly distinct mechanisms. Moreover, because the concentration of endogenous TNF in the culture medium was not determined, the 20 ng/mL recombinant TNF treatment should be regarded as an experimental perturbation rather than as an attempt to reproduce the endogenous TNF concentration following TWEAK treatment. TNF treatment also significantly reduced *E-cadherin*, *Lgr5*, and *Ki-67* mRNAexpression. These changes suggest possible effects on intercellular adhesion, stem cell-associated properties, and proliferative activity. They are also broadly consistent with previous reports showing that TNF can impair epithelial integrity and barrier function during intestinal inflammation [25,26,27,28,38,39].

The reduction in E-cadherin expression after TNF treatment may contribute to changes in epithelial organization. Altered E-cadherin expression has been associated with epithelial structural abnormalities and EMT-like changes in several experimental systems [40,41,42]. However, because EMT-related markers and cellular phenotypes were not examined in the present study, it would be premature to conclude that TNF induced an EMT-like process in these organoids.

TNF treatment reduced the number of MUC2-positive cells and the PAS-positive area while increasing the relative proportion of AB-positive acidic mucin. Importantly, the reduction in MUC2-positive cells after TNF treatment does not necessarily indicate a reduction in the absolute number of goblet cells. This change could reflect a decrease in goblet cell abundance, reduced MUC2 expression in surviving cells, or both. Although *Klf4* and *Spdef* were examined as goblet cell differentiation-associated genes, these markers alone are insufficient to determine goblet cell abundance or differentiation state. In addition, cell-death pathways, including apoptosis and necroptosis, were not evaluated. Therefore, these possibilities cannot be distinguished in the present study. These findings suggest that TNF treatment reduces the number of MUC2-positive cells and overall mucin content while altering the relative composition of the remaining mucin. PGM34 staining also significantly increased following TNF treatment, consistent with the tendency toward increased PGM34 staining observed after TWEAK treatment. Acidic mucins include sialomucins and sulfomucins, which are distinguished by their glycosylation patterns [43]. Increased sulfomucin has been reported in the small intestine during parasitic infection, and enhanced mucin sulfation has been associated with increased Tnf mRNA expression in some bacterial infection models [22,44]. These observations are consistent with the possibility that inflammatory signaling influences mucin glycosylation. However, whether the changes observed in the present study represent a protective adaptation, a consequence of epithelial stress, or a shift in goblet cell phenotype remains unclear.

TNF treatment also produced a tendency toward increased *Muc5ac* mRNA expression. MUC5AC is normally expressed at low levels in the small intestine but can be induced under inflammatory or injury-associated conditions [45]. Increased intestinal MUC5AC expression has also been reported in patients with inflammatory bowel disease [46,47]. More broadly, MUC2 and MUC5AC expression patterns have been used to distinguish intestinal and gastric mucin phenotypes in gastrointestinal pathology. In gastric lesions and intestinal metaplasia, MUC2 is associated with an intestinal phenotype, whereas MUC5AC is associated with a gastric phenotype, together with differentiation-associated markers such as CDX2 [8]. From this perspective, the tendency toward increased Muc5ac mRNA expression observed following TNF treatment may reflect a change in the epithelial mucin phenotype rather than simply a quantitative change in mucin expression. However, because the increase in Muc5ac expression was not statistically significant, MUC5AC protein expression was not evaluated, and additional lineage or differentiation markers relevant to such a phenotypic change were not examined, this interpretation should be regarded as preliminary.

To further examine the contribution of TNF to TWEAK-associated responses, we evaluated TWEAK treatment in the presence of a TNF inhibitor. TNF inhibition increased *Lgr5* and *Ki-67* expression regardless of the presence of TWEAK, suggesting that endogenous TNF signaling may restrain the expression of stem cell- and proliferation-associated genes under these culture conditions [27,48]. Because the anti-TNF antibody also affected gene expression in the absence of TWEAK, these effects may partly reflect neutralization of basal endogenous TNF activity rather than specific blockade of the modest TWEAK-associated increase in *Tnf* mRNA expression. Since TNF protein concentrations in the culture medium were not measured, the relative contributions of basal and TWEAK-associated TNF activity cannot be distinguished in the present study. Combined treatment with TWEAK and the TNF inhibitor increased *E-cadherin* and *Muc2* mRNA expression, whereas the inhibitor alone did not increase *Muc2* mRNA expression. These findings suggest that TNF inhibition modifies the epithelial response to TWEAK, particularly with respect to *E-cadherin* and *Muc2 mRNA* expression.

In the histological analyses, combined treatment was associated with a tendency toward increased MUC2-positive cell numbers, a significant increase in PAS-positive mucin, and a tendency toward increased PGM34 staining, together with a significant decrease in the proportion of AB-positive acidic mucin. These results suggest that the epithelial response to TWEAK differs depending on the status of TNF signaling. Nevertheless, the present experiments do not establish whether TWEAK directly enhances goblet cell differentiation or mucin production when TNF is inhibited. Additional studies examining lineage markers, mucin secretion, and pathway-specific signaling will be required to clarify the underlying mechanisms.

Notch signaling is an important regulator of intestinal epithelial lineage allocation, and its inhibition promotes differentiation toward secretory lineages, including goblet cells [49,50]. Although interactions between TWEAK/Fn14 and Notch signaling were not examined in the present study, it will be of interest to determine whether changes in Notch-associated pathways contribute to the goblet cell and mucin-related responses observed here.

This study has several limitations. First, the organoid model lacks immune cells, stromal cells, neural components, and microbiota, all of which influence epithelial differentiation and mucus barrier function. Therefore, the findings reflect epithelial-intrinsic responses under simplified culture conditions and may not fully represent the responses occurring in vivo. Second, Fn14-specific inhibition or genetic manipulation was not performed. Consequently, although TWEAK was used as the Fn14 ligand, the extent to which the observed responses were specifically mediated through Fn14 cannot be fully determined. Third, several mucin-related changes were observed only as trends, and mucin secretion, glycosylation, and functional barrier properties were not directly measured. Fourth, an isotype-matched control antibody was not included in the experiments using the neutralizing anti-TNF antibody. Therefore, nonspecific effects associated with antibody treatment cannot be completely excluded, and the effects observed following anti-TNF antibody treatment should be interpreted as suggesting the involvement of TNF signaling rather than providing definitive evidence that these effects were solely attributable to TNF neutralization. Fifth, the number of organoid culture samples was relatively small for some gene-expression analyses (n = 3–6), which may limit the statistical power and robustness of these findings. In addition, although organoid cultures from individual donor mice were established separately and were not pooled, more than one culture sample and multiple organoids were analyzed from some donor mice. Therefore, the numbers of culture samples and individual organoids should not be interpreted as equivalent to the number of independent donor animals. Future studies with donor mouse-level replication and statistical analysis will be important to confirm these findings. Finally, TNF was assessed only at the mRNA level, and TNF protein concentrations in the culture medium were not measured. Therefore, the present findings do not establish increased TNF protein production or secretion following TWEAK treatment.

## 5. Conclusions

In conclusion, the present findings suggest that TWEAK treatment was associated with goblet cell-related responses and mucin-associated changes in small intestinal epithelial organoids. Fn14 staining was preferentially observed toward the mucin-facing region of MUC2-positive cells, and TWEAK treatment reduced *Muc2* mRNA expression while increasing Tnf mRNA expression. TNF treatment reproduced some, but not all, of the changes observed following TWEAK treatment and additionally reduced the expression of genes associated with epithelial adhesion, stem cell function, and proliferation. The distinct effects of TWEAK and TNF on MUC2-positive cell number further suggest that their actions are only partially overlapping. Moreover, TNF inhibition modified the epithelial response to TWEAK, particularly with respect to *E-cadherin*, *Muc2*, and mucin-associated staining patterns. Together, these findings suggest that TNF may contribute to a subset of TWEAK-associated epithelial responses, while TWEAK and TNF may also affect goblet cell-associated features through partly distinct mechanisms. These findings provide a basis for further studies examining the relationship between TWEAK-associated epithelial responses, TNF signaling, MUC2-positive cells, and mucin-associated features in the small intestinal epithelium.

## Data Availability

The data presented in this study are available from the corresponding author upon reasonable request.

## Supplementary Materials

The following supporting information can be downloaded at: https://www.mdpi.com/article/10.3390/cells15171541/s1, Figure S1: Effect of TWEAK treatment on goblet cell differentiation-associated genes in small intestinal epithelial organoids. Relative *Klf4* and *Spdef* mRNA expression measured by quantitative real-time PCR (*n* = 5–6). Organoid cultures were derived from four donor mice per group. A total of six culture samples per group were analyzed, with one or two culture samples obtained from each donor mouse. These culture samples were analyzed in two independent experiments. Data are presented as the mean ± standard error of the mean (SEM). Statistical comparisons were performed using an unpaired Welch’s *t*-test; Figure S2: Assessment of inflammatory cytokine mRNA expression in mouse small intestinal epithelial organoids by qRT-PCR and agarose gel electrophoresis. (A) The expression of *Interferon gamma* (*Ifng*), *Interleukin-6* (*Il6*), and *Interleukin 1 beta* (*Il1b*) was examined by quantitative real-time PCR in control, TWEAK-treated, TNF-treated, TNF inhibitor-treated (Anti-TNF), and TWEAK plus TNF inhibitor (Anti-TNF)-treated organoids (n = 5–6 per group). PCR products were subsequently analyzed by agarose gel electrophoresis. No detectable amplification products corresponding to Ifng or Il6 were observed in any of the experimental groups. For Il1b, bands were observed; however, their sizes did not correspond to the expected amplicon size (148bp: Dotted-line section) and were therefore considered nonspecific amplification products. Gapdh was consistently amplified as an internal control. These results indicate that specific amplification of Ifng, Il6, and Il1b was not detected under the experimental conditions used. Original raw agarose gel images are shown.

## Abbreviations

The following abbreviations are used in this manuscript:

AB-PAS	Alcian blue–periodic acid–Schiff
ANOVA	Analysis of variance
cDNA	Complementary DNA
DAB	3,3′-Diaminobenzidine
DAPI	4′,6-Diamidino-2-phenylindole
DMEM/F-12	Dulbecco’s modified Eagle medium/nutrient mixture F-12
D-PBS	Dulbecco’s phosphate-buffered saline
EDTA	Ethylenediaminetetraacetic acid
EGF	Epidermal growth factor
EMT	Epithelial–mesenchymal transition
Fn14	Fibroblast growth factor-inducible 14
HEPES	4-(2-Hydroxyethyl)-1-piperazineethanesulfonic acid
HRP	Horseradish peroxidase
IBD	Inflammatory bowel disease
PAS	Periodic acid–Schiff
PBS	Phosphate-buffered saline
qRT-PCR	Quantitative reverse-transcription polymerase chain reaction
SEM	Standard error of the mean
TNF	Tumor necrosis factor
TWEAK	Tumor necrosis factor-like weak inducer of apoptosis
