# Supplementary material for: TWEAK/Fn14 Signaling Modulates Goblet Cell-Associated Mucin Responses in Mouse Small Intestinal Epithelial Organoids
Source: Cells. 2026 Aug 26;15(17):1541. doi: 10.3390/cells15171541 (PMC13565329; doi:10.3390/cells15171541)
Supplement: Supplementary file 1 [file cells-15-01541-s001.zip › Figure S2.pdf]

Supplementary material

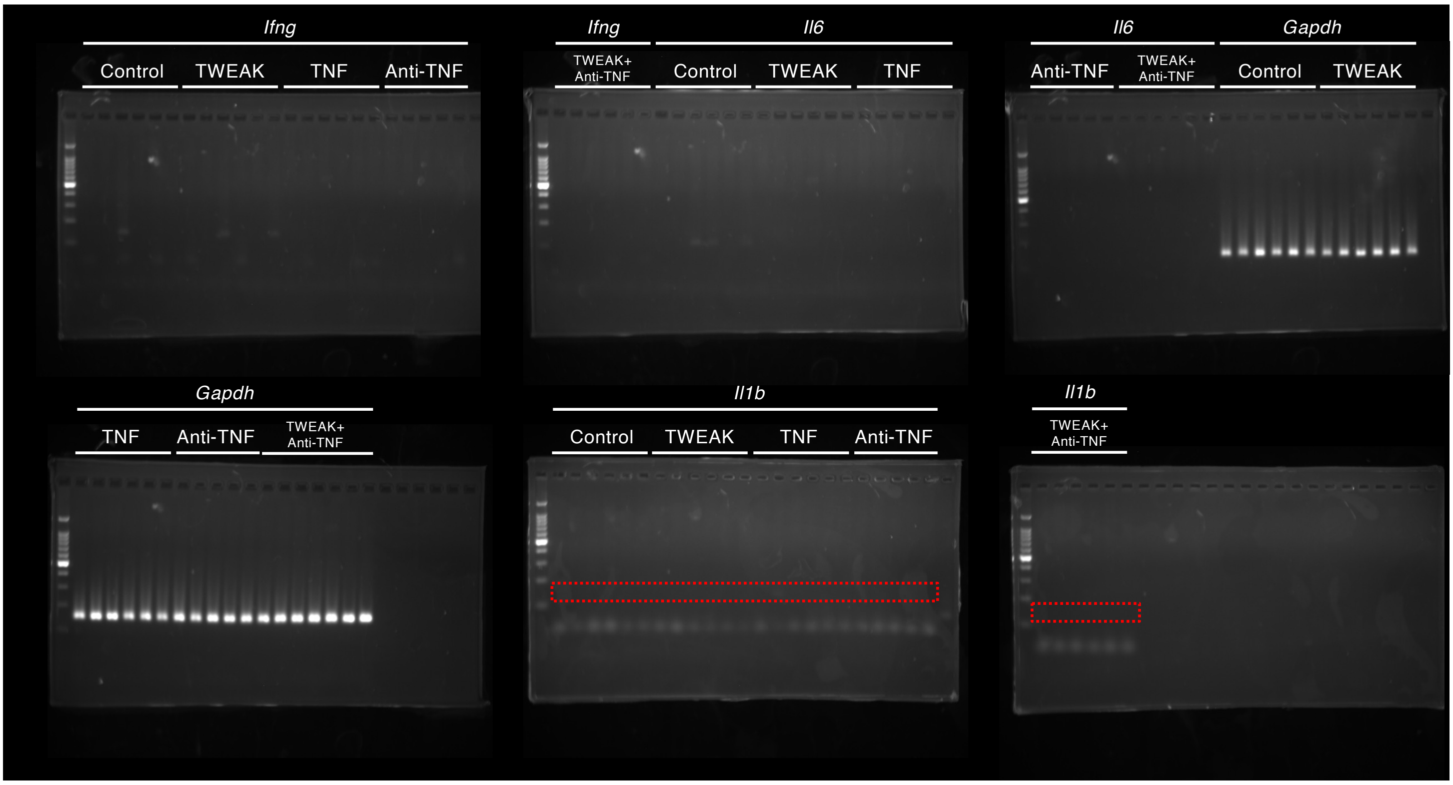

Figure S2. Assessment of inflammatory cytokine mRNA expression in mouse small intestinal epithelial organoids by qRT-PCR and agarose gel electrophoresis

(A) The expression of *Interferon gamma (Ifng), Interleukin-6 (Il6),* and *Interleukin 1 beta (Il1b)* was examined by quantitative real-time PCR in control, TWEAK-treated, TNF-treated, TNF inhibitor-treated (Anti-TNF), and TWEAK plus TNF inhibitor (Anti-TNF)-treated organoids (*n = 5–6 per group*). PCR products were subsequently analyzed by agarose gel electrophoresis. No detectable amplification products corresponding to Ifng or Il6 were observed in any of the experimental groups. For Il1b, bands were observed; however, their sizes did not correspond to the expected amplicon size (148bp:Dotted-line section) and were therefore considered nonspecific amplification products. Gapdh was consistently amplified as an internal control. These results indicate that specific amplification of Ifng, Il6, and Il1b was not detected under the experimental conditions used. Original raw agarose gel images are shown.
