# Supplementary material for: TWEAK/Fn14 Signaling Modulates Goblet Cell-Associated Mucin Responses in Mouse Small Intestinal Epithelial Organoids
Source: Cells. 2026 Aug 26;15(17):1541. doi: 10.3390/cells15171541 (PMC13565329; doi:10.3390/cells15171541)
Supplement: Supplementary file 1 [file cells-15-01541-s001.zip › Figure S1.pdf]

Supplementary material

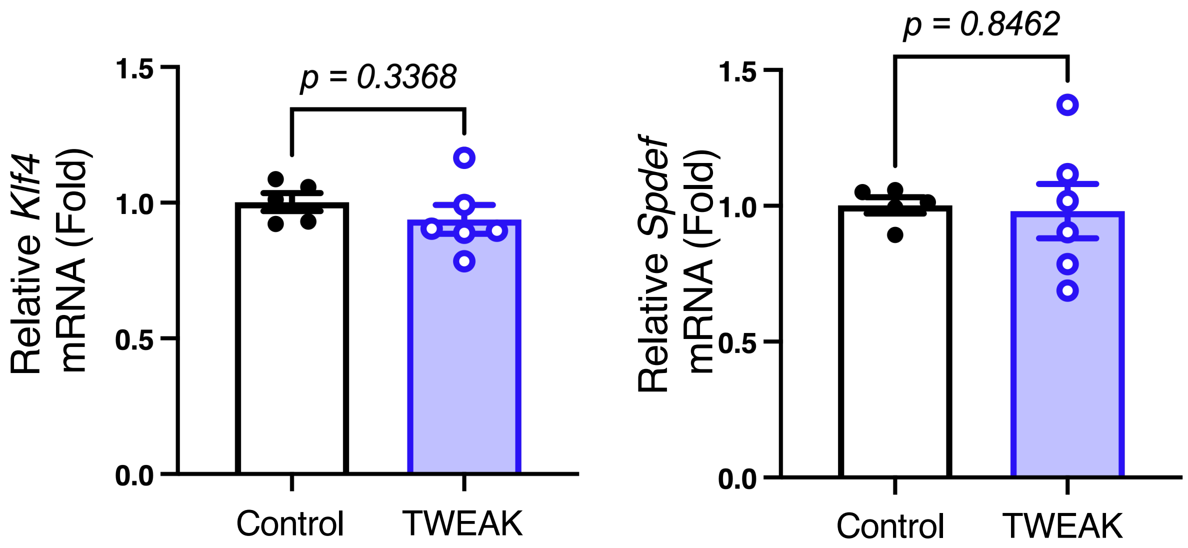

Figure S1. Effect of TWEAK treatment on goblet cell differentiation-associated genes in small intestinal epithelial organoids.

Relative *Klf4* and *Spdef* mRNA expression measured by quantitative real-time PCR (*n* =5-6). Organoid cultures were derived from four donor mice per group. A total of six culture samples per group were analyzed, with one or two culture samples obtained from each donor mouse. These culture samples were analyzed in two independent experiments. Data are presented as the mean ± standard error of the mean (SEM). Statistical comparisons were performed using an unpaired Welch's t-test.
